# Mother–egg stable isotope conversions and effects of lipid extraction and ethanol preservation on loggerhead eggs

**DOI:** 10.1093/conphys/cou049

**Published:** 2014-10-30

**Authors:** Temma J. Kaufman, Mariela Pajuelo, Karen A. Bjorndal, Alan B. Bolten, Joseph B. Pfaller, Kristina L. Williams, Hannah B. Vander Zanden

**Affiliations:** 1 Archie Carr Center for Sea Turtle Research and Department of Biology, University of Florida, PO Box 118525, Gainesville, FL 32611, USA; 2 Caretta Research Project, PO Box 9841, Savannah, GA 31412, USA

**Keywords:** Albumen, carbon isotopes, *Caretta caretta*, nitrogen isotopes, yolk

## Abstract

Carbon and nitrogen stable isotope (δ^13^C and δ^15^N) analysis has been used to elucidate foraging and migration behaviours of endangered sea turtle populations. Isotopic analysis of tissue samples from nesting females can provide information about their foraging locations before reproduction. To determine whether loggerhead (*Caretta caretta*) eggs provide a good proxy for maternal isotope values, we addressed the following three objectives: (i) we evaluated isotopic effects of ethanol preservation and lipid extraction on yolk; (ii) we examined the isotopic offset between maternal epidermis and corresponding egg yolk and albumen tissue δ^13^C and δ^15^N values; and (iii) we assessed the accuracy of foraging ground assignment using egg yolk and albumen stable isotope values as a proxy for maternal epidermis. Epidermis (*n* = 61), albumen (*n* = 61) and yolk samples (*n* = 24) were collected in 2011 from nesting females at Wassaw Island, GA, USA. Subsamples from frozen and ethanol-preserved yolk samples were lipid extracted. Both lipid extraction and ethanol preservation significantly affected yolk δ^13^C, while δ^15^N values were not altered at a biologically relevant level. The mathematical corrections provided here allow for normalization of yolk δ^13^C values with these treatments. Significant tissue conversion equations were found between δ^13^C and δ^15^N values of maternal epidermis and corresponding yolk and albumen. Finally, the consistency in assignment to a foraging area was high (up to 84%), indicating that these conversion equations can be used in future studies where stable isotopes are measured to determine female foraging behaviour and trophic relationships by assessing egg components. Loggerhead eggs can thus provide reliable isotopic information when samples from nesting females cannot be obtained.

## Abstract (precis)

Loggerhead egg stable isotopic composition can be used as a proxy for mother values as a result of the strong correlation between mother and egg tissue. Also, the significant effects of lipid extraction and ethanol preservation on carbon isotope values can be accounted for using mathematical corrections.

## Introduction

Six of the seven sea turtle species are considered vulnerable, endangered or critically endangered according to the International Union for Conservation of Nature Red List, while there are not adequate data to assess the status of the seventh species (IUCN, 2014). The status of loggerhead sea turtles (*Caretta caretta*) is endangered. Many of the distinct population segments are declining as a result of human disturbances at nesting beaches and incidental capture in fisheries (Conant *et al.*, 2009). However, because loggerhead sea turtles spend the majority of their lives in the marine environment, it can be difficult to identify where and how they are feeding in order to mitigate potential threats.

Stable isotope analysis has become an increasingly useful technique in both marine and terrestrial ecological studies. In animals, carbon (δ^13^C) and nitrogen (δ^15^N) stable isotopes are assimilated through the diet, and their values reflect the animal's foraging habitats and trophic position (Vander Zanden and Rasmussen, 2001; Post, 2002). In the marine environment, stable isotope values are also influenced by spatial variation in the base of the food web and can therefore reflect the location of foraging (Graham *et al.*, 2010; McMahon *et al.*, 2013). Analysis of tissue isotopic values has been useful in studying the behavioural and ecological patterns of many migratory marine organisms (reviewed by Ramos and González-Solís, 2012; McMahon *et al.*, 2013). More specifically, in sea turtles, tissue samples collected from nesting females can represent the diet and foraging patterns prior to the reproductive period. Stable isotope analysis has been used to determine foraging locations of nesting adult loggerheads (Hatase *et al.*, 2002; Ceriani *et al.*, 2012; Pajuelo *et al.*, 2012) and link foraging site preference with reproductive output (Zbinden *et al.*, 2011; Hatase *et al.*, 2013; Vander Zanden *et al.*, 2014a). Monitoring the trends in foraging area origin over several years and combining abundance trends with the reproductive data allows us to better understand the demographic parameters that are affecting nesting aggregations (Vander Zanden *et al.*, 2014a).

Sea turtle research has been biased toward nesting females as a result of their accessibility at rookeries, and therefore, counting females and nests provides a practical means for assessing sea turtle population status and size. After nesting, however, it becomes difficult to sample a sea turtle until the next nesting event. For most sea turtle species, females return to nest every 2–4 years (Broderick *et al.*, 2003). Furthermore, in many sea turtle monitoring programmes, such as the Florida Fish and Wildlife Conservation Commission Index Nesting Beach Survey (Witherington *et al.*, 2009), nightly patrols are not conducted, and therefore females are not encountered, but rather emergence and nesting events are assessed each morning based on track characteristics. Even when nightly patrols are conducted, not every female may be encountered during nesting. Therefore, sampling from a large number of sea turtles at a nesting beach may not always be possible. The use of offspring tissues for stable isotope analysis may increase the sample size to study the adult female population if there is a reliable relationship between maternal and offspring stable isotope values.

Offspring tissues are derived from maternal resources, and therefore, an isotopic relationship between maternal and offspring tissues has been observed in multiple species (Jenkins *et al.*, 2001; Sare *et al.*, 2005; Caut *et al.*, 2008; McMeans *et al.*, 2009; Vaudo *et al.*, 2010; Zbinden *et al.*, 2011). We follow the recommendation of Vander Zanden *et al.* (2014b) to refer to the comparison of isotopic values between two tissues of the same organism (including offspring tissues derived from the mother) as tissue-to-tissue conversions rather than discrimination factors, which should be reserved for the isotopic difference between tissues and diet. The offset between maternal and offspring isotopic values can be calculated as the mean difference between the two tissue types or by using linear regression, but regression is preferable because single-value conversion factors can lead to spurious results if the slope of the relationship is different from one (Vander Zanden *et al.*, 2014b). Statistically significant correlations have been observed between nesting female sea turtle and offspring tissues in leatherbacks (*Dermochelys coriacea*) and loggerheads (Caut *et al.*, 2008; Zbinden *et al.*, 2011; Frankel *et al.*, 2012; Ceriani *et al.*, 2014), although none of these studies has considered albumen when evaluating mother–offspring relationships nor have they examined the effects of common preservation and lipid extraction techniques on egg yolk.

Egg component samples are typically preserved in ethanol or frozen until isotope analysis is performed. Ethanol preservation and freezing have commonly been used for sea turtle eggs (Hamann *et al.*, 2002; Caut *et al.*, 2008; Zbinden *et al.*, 2011) and eggs from other taxa (Gloutney and Hobson, 1998; Elliott *et al.*, 2014). Freezing samples, however, requires access to a freezer during storage and subsequent transport. When freezers are not available at field sites, ethanol preservation can be more convenient for storing eggs after collection at nesting beaches. While freezing has often been considered a safe method of preservation (Gloutney and Hobson, 1998; Barrow *et al.*, 2008; Elliott *et al.*, 2014), preservation of sea turtle egg tissues in ethanol has not been validated.

Prior to stable isotope analysis, samples with elevated lipid content, such as egg yolks, are typically lipid extracted, because lipids have considerably lower δ^13^C values than other cell components, such as proteins and carbohydrates (DeNiro and Epstein, 1977); therefore, tissues typically have higher δ^13^C values after lipid extraction. Given that lipid content can vary between organisms and tissue types, lipid extraction negates any resulting biases of lipid content on δ^13^C analysis (Post *et al.*, 2007). Without lipid extraction, variation in isotopic values due to differences in lipid content could be mistaken for location or diet changes. However, lipid extraction may affect δ^15^N values as well (Sweeting *et al.*, 2005; Doucette *et al.*, 2010; Oppel *et al.*, 2010a; Elliott *et al.*, 2014). The effects on δ^15^N values can be avoided by analysing each sample twice; once before lipid extraction for δ^15^N values and again after lipid extraction for δ^13^C values. This doubles the cost and time for analysis.

To circumvent lipid extraction entirely, mathematical corrections have been used to normalize lipid content in many cases (Sweeting *et al.*, 2005; Post *et al.*, 2007; Elliott *et al.*, 2014). Although normalization has been used in a wide range of species and tissues, these mathematical corrections have not previously been determined for marine turtle yolks. Mathematical normalizations for lipid extraction of avian yolks have been determined, but these corrections may not be applicable to other species (Doucette *et al.*, 2010; Elliott *et al.*, 2014). Additionally, the aquatic animal meta-analysis to provide generalized lipid normalization equations included few tissue samples with ≥25% lipid content (Post *et al.*, 2007), and loggerhead egg yolks may contain up to 25% lipids (Alava *et al.*, 2006). Therefore, the mathematical corrections suggested by Post *et al.* (2007) may not be applicable to sea turtle yolks as a result of the high lipid content, and the recommendations made for lipid normalization in that study do not apply for samples that have preserved in ethanol.

The objectives of this study were 3-fold. First, we evaluated the effects of ethanol preservation and lipid extraction on the δ^13^C and δ^15^N values of loggerhead yolk. Quantifying changes in isotopic values due to these treatments is necessary to ensure accurate mother–egg conversions. Preservation in 95% ethanol was not expected to cause significant changes in δ^13^C and δ^15^N values, because previous studies have found no isotopic difference between frozen and ethanol-preserved tissue samples (Gloutney and Hobson, 1998; Barrow *et al.*, 2008). As indicated earlier, lipid extraction was expected to cause an increase in δ^13^C values. We also predicted that lipid extraction would not have a significant effect on δ^15^N values, because lipid extraction by non-polar solvents should not affect δ^15^N values (Dobush *et al.*, 1985; Doucette *et al.*, 2010; but see Logan and Lutcavage, 2008). Second, we used linear regression to provide reliable conversion equations between δ^13^C and δ^15^N values of adult female loggerhead sea turtles and their egg components (albumen and yolk). Third, we applied the mother–egg conversion equations to test the accuracy of determining the foraging area origin of the nesting female by using the stable isotope values of egg yolk and albumen as a proxy for maternal tissues. These results will be useful in future studies that use stable isotopes to determine female foraging behaviour and trophic relationships by assessing egg components.

## Materials and methods

### Sample collection and preservation

During the 2011 nesting season (May to July), skin was sampled from nesting loggerheads (*n* = 61) at Wassaw Island, GA, USA (31.89°N, 80.97°W). Skin samples were collected using a sterile 6 mm biopsy punch (Miltex) in the ‘shoulder’ area between the neck and front flipper. Skin samples were preserved in 70% ethanol and stored at room temperature for almost 2 months before stable isotope analysis. Preservation in 70% ethanol does not significantly affect turtle skin stable isotope values (Barrow *et al.*, 2008).

One egg was collected from the corresponding female's nest and separated into yolk and albumen. Intra- and inter-clutch variation in loggerhead egg yolk stable isotope ratios is minimal (Hatase *et al.*, 2002; Zbinden *et al.*, 2011; Ceriani *et al.*, 2014); therefore, using one egg from each female is likely to be representative of the stable isotope values that would be observed in eggs from within a clutch and within the nesting season. The eggs used in this study were sacrificed for a separate genetic study (B. M. Shamblin, unpublished data); no sea turtle embryos were sacrificed for this study. Egg yolk samples were frozen at −20°C, and subsamples of 24 egg yolks were preserved in 95% ethanol prior to freezing the remainder of the sample. The concentration of ethanol was selected as 95% because we thought that a concentration of 70% ethanol would become too dilute with a liquid-based sample. Frozen samples were stored for up to 6 months prior to analysis, and ethanol-preserved samples were stored for up to 15 months. All albumen samples were frozen immediately after collection at −20°C for up to 4 months before analysis. Albumen was not preserved in ethanol owing to the liquid nature of the sample. The sample size for yolks (*n* = 24) was smaller than that of the albumen samples (*n* = 61) because we did not subset all of the yolks for preservation in ethanol due to the limitations of the time the field technicians could devote to this sampling. Therefore, we limited our analyses to only the yolks that had paired frozen and ethanol-preserved samples.

Egg and epidermis samples were typically taken at the earliest observed nesting event during the season. When an egg was not collected on the same date as the skin sample, egg samples were used from the next closest sampling date, resulting in a maximum of 38 days between skin and egg collection. This occurred when a female was observed and a skin sample was obtained, but then she failed to nest and an egg sample could not be obtained until the subsequent nesting event at which she was observed. Maternal epidermis was collected 2–23 days prior to the collection of three yolk samples and 2–38 days prior to the collection of six albumen samples.

### Sample preparation and analysis

Skin samples were cleaned by a distilled water rinse and then wiped with isopropyl alcohol swabs. The skin surface (epidermis) was separated from the dermis and then homogenized using a scalpel blade before drying at 60°C for 24–48 h. Samples of epidermis were not lipid extracted before stable isotope analysis, because lipid extraction does not affect the stable isotope values of loggerhead epidermis (Vander Zanden *et al.*, 2014a).

**Figure 1:**
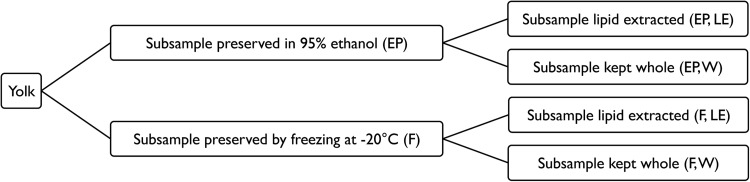
Each loggerhead yolk (*n* = 24) in this study was subsampled, and isotopic values were analysed after undergoing four different treatments to evaluate the isotopic effects of ethanol preservation and lipid extraction.

Portions of the yolk and albumen samples were pipetted into weigh boats and dried at 60°C for 24–48 h. Prior to pipetting, ethanol-preserved yolk samples were shaken and mixed well to obtain a homogeneous portion of the liquid. To test the effect of lipid extraction on frozen and ethanol-preserved egg yolk samples, subsamples of the frozen and ethanol-preserved yolks were lipid extracted before stable isotope analysis (Fig. 1). We refer to yolks that were not lipid extracted as whole yolks, regardless of the preservation method.

Lipid extraction was performed using an accelerated solvent extractor (ASE300) with petroleum ether solvent for three consecutive cycles consisting of 5 min of heating to 100°C and pressurization to 1500 psi, 5 min static, purging, and then flushing with additional solvent. This non-polar solvent was selected because polar solvents tend to extract significant amounts of non-lipid material and may be more likely to affect δ^15^N values (Dobush *et al.*, 1985; Doucette *et al.*, 2010).

Albumen was not lipid extracted in this study due to limited sample size and low lipid content. Additionally, in avian egg stable isotope studies, albumen is not typically lipid extracted (Hobson, 1995; Bond *et al.*, 2007; Polito *et al.*, 2009; Quillfeldt *et al.*, 2009; Oppel *et al.*, 2010b). The C:N ratio is strongly correlated with the lipid content of animal tissue, and lipid extraction or normalization is recommended when the C:N ratio exceeds 3.5 (Post *et al.*, 2007). The mean C:N ratio of loggerhead albumen was only slightly higher than the recommended cut-off (mean C:N = 3.68 ± 0.32).

Approximately 0.5–0.6 mg of each sample was loaded into a sterile 4 mm × 6 mm tin capsule and analysed for the percentage of carbon, percentage of nitrogen, δ^13^C and δ^15^N values. Analyses were performed using one of the following three systems in the Department of Geological Sciences at the University of Florida: (i) a Thermo Finnigan DeltaPlus XL isotope mass spectrometer with a ConFlo III interface linked to a Costech ECS 4010 elemental analyser; (ii) a Finnigan-MAT 252 isotope ratio mass spectrometer with a ConFlo II interface linked to a Carlo Erba NA 1500 CNS elemental analyser; or (iii) a Thermo Electron DeltaV Advantage isotope ratio mass spectrometer coupled with a Conflo II interface linked to a Carlo Erba NA 1500 CNS elemental analyser. Stable isotope abundances were expressed in delta notation and defined as parts per thousand (‰) relative to the standard, as follows:

$$\delta =\frac{R_{\mathrm{sample}}}{R_{\mathrm{standard}}}-1$$

where *R*_sample_ and *R*_standard_ are corresponding ratios of heavy to light isotopes (^13^C:^12^C and^15^N:^14^N) in the sample and international standard. The standard for^13^C used was Vienna Pee Dee Belemnite, while atmospheric N_2_ was used as the standard for^15^N. All analyses used the reference material USGS40 (l-glutamic acid) as a calibration standard. The standard deviation of this reference material was 0.05‰ for δ^13^C and 0.16‰ for δ^15^N (*n* = 63). Repeated measurements of a laboratory reference material, loggerhead scute, were used to ensure consistency among the results from the different instruments and examine variance in a homogeneous sample with similar isotopic composition to the samples in this study. The standard deviation for this laboratory reference material was 0.12‰ for δ^13^C and 0.27‰ for δ^15^N (*n* = 18). The C:N ratio was calculated by dividing the percentage of carbon by the percentage of nitrogen.

### Statistical analysis

A linear mixed-effects model was performed to determine the interaction and main effects of ethanol preservation or lipid extraction on δ^13^C and δ^15^N values. In the mixed-effects model, preservation method and lipid extraction were considered to be fixed factors, while individual turtles were random factors. Tukey's *post hoc* tests were used to make pair-wise comparisons among treatments (see Fig. 1 for a diagram of the experimental design). The sample size of yolk (*n* = 24) was smaller than that of albumen (*n* = 61), so a power analysis was conducted to determine the minimal detectable difference in δ^13^C values and δ^15^N values in performing pair-wise analyses of yolk samples using a power of 0.9 and assuming a variance among samples equal to the maximal variance observed within clutches as measured by Ceriani *et al.* (2014) (δ^13^C σ^2^ = 0.53‰, δ^15^N σ^2^ = 0.47‰). This resulted in a minimal detectable difference of 0.19‰ in δ^13^C values and 0.15‰ in δ^15^N values, and thus, confirmed that our sample size was adequate for detecting biologically relevant differences among yolk treatments. Linear regressions were performed to determine mathematical corrections for lipid extraction and ethanol preservation for δ^13^C values.

Ratios of C:N in untreated samples have been a reliable predictor for the change in δ^13^C values as a result of lipid extraction (represented by Δδ^13^C), as the C:N ratio acts as a proxy for lipid content because nitrogen abundance is high in proteins and low in lipids (Post *et al.*, 2007); therefore, C:N ratios before and after lipid extraction were compared using Student's paired *t*-tests. To determine whether C:N ratios could be used for normalization, linear regressions were performed between whole yolk C:N ratios and the difference in δ^13^C values after lipid extraction (Δδ^13^C = δ^13^C of lipid-extracted yolk minus δ^13^C of whole yolk).

The relationship between maternal (epidermis) and egg (yolk or albumen) stable isotope values was also approximated using linear regressions for both δ^13^C and δ^15^N values. Comparisons were made between epidermis and the four yolk treatment combinations. We report mother–offspring conversions as linear regressions rather than a mean difference between maternal epidermis and egg components. When the slopes in the regression equations are not equal to one, the conversion factor between mother and offspring will vary over a range of isotope values. Therefore, applying a single-value conversion factor may yield less reliable results than applying the regression equations (Vander Zanden *et al.*, 2014b). All statistical analyses were performed using the program R (R Core Team, 2013) with an α level of 0.05.

### Using egg components to determine foraging area

To assess the reliability of the relationships between maternal epidermis and egg components, we predicted maternal epidermis isotopic values from egg yolk and albumen isotopic values using the conversion equations obtained in this study, and determined the turtles' foraging areas based on these predicted maternal isotopic values. Turtles were assigned to one of the three distinct foraging regions in the Northwest Atlantic (NWA) using δ^13^C and δ^15^N values jointly [the Mid-Atlantic Bight (MAB), the South Atlantic Bight (SAB) and the subtropical NWA (SNWA)] using a discriminant analysis created with training data from 60 adult loggerheads with known foraging locations (see Pajuelo *et al.*, 2012 for detailed methodology).

Similar to previous studies, only assignments with a probability of group membership ≥80% were considered (Pajuelo *et al.*, 2012; Seminoff *et al.*, 2012; Vander Zanden *et al.*, 2014a). Using this threshold provides an 8-fold improvement over random odds (Wunder, 2012). The assignment process was performed twice for each individual. First, epidermis isotopic data from mother loggerheads sampled in our study were used to assign individual turtles to a foraging area. If mother assignments did not meet the threshold, the egg component conversion was not tested. Next, those individuals were assigned using the predicted epidermis isotopic values based on isotopic data for egg components. Again, some egg samples did not meet the selected probability threshold. The accuracy of assignment using the converted egg component isotopic values was obtained as the percentage of individuals meeting the probability threshold that were assigned to the same foraging area as when the maternal epidermis isotopic values were used.

## Results

### Effect of ethanol preservation and lipid extraction

**Figure 2:**
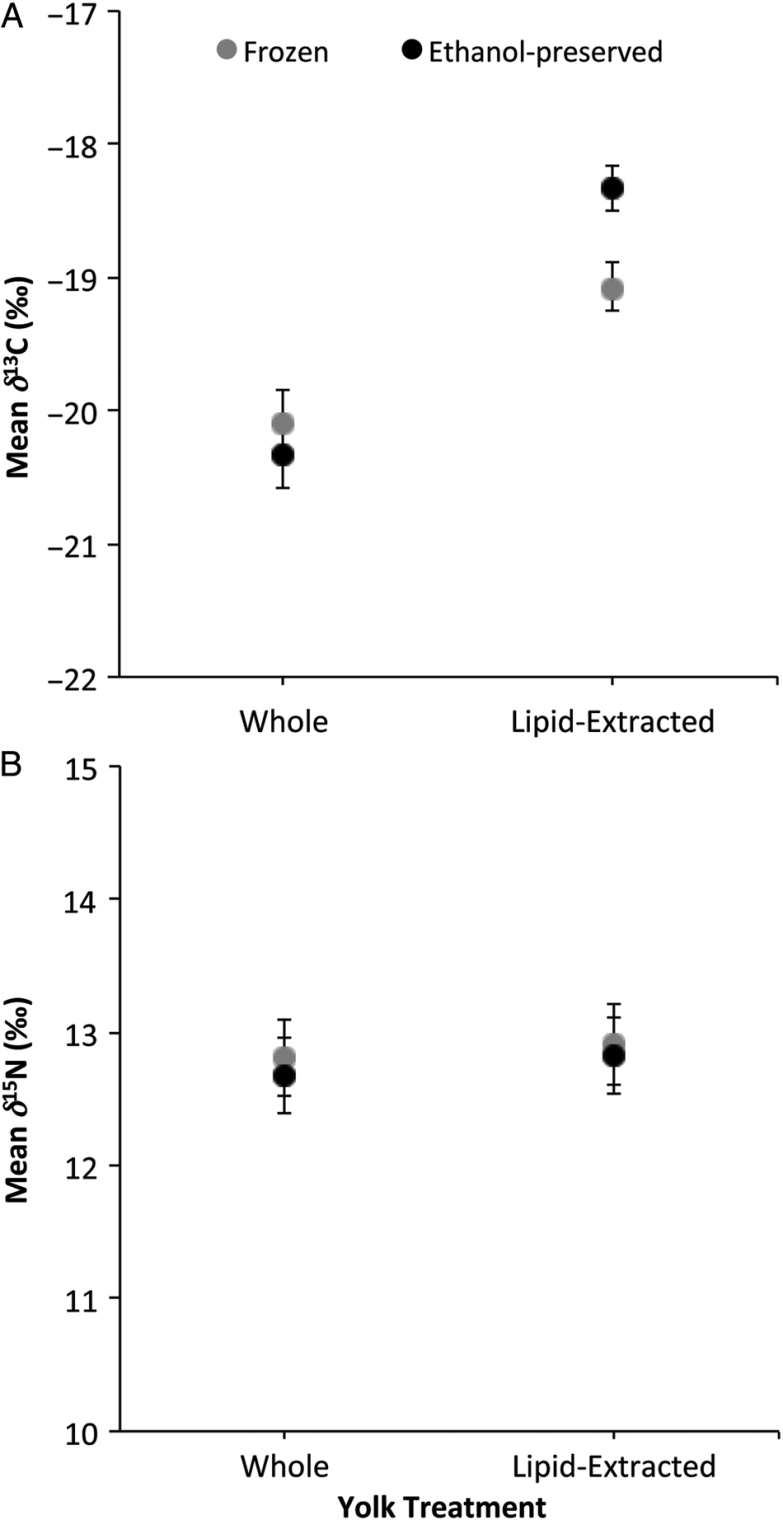
Mean ± SEM δ^13^C (**A**) and δ^15^N values (**B**) of whole (not lipid-extracted) and lipid-extracted yolks with two preservation treatments, frozen and ethanol preserved. Lipid extraction and ethanol preservation exhibited an interaction effect for δ^13^C values but not for δ^15^N values.

The linear mixed-effects model indicated that there was a significant interaction between lipid extraction and the preservation method in δ^13^C values (*P* < 0.0001), but not in δ^15^N values (*P* = 0.70; Fig. 2); therefore, the main effects cannot be interpreted independently for the δ^13^C values (Table 1). Instead, Tukey's *post hoc* comparisons indicated that there was no effect of ethanol preservation on the δ^13^C values of whole samples (Table 2). However, there was a difference in δ^13^C values of freezing and ethanol treatments on samples that were lipid extracted, such that samples that were preserved in ethanol and lipid extracted had significantly higher δ^13^C values (mean difference = 0.75‰) than those that were frozen and lipid extracted (Table 2). The effect of ethanol preservation on δ^13^C values of lipid-extracted yolks can be estimated with the normalization equations in Table 3. There was no significant difference in δ^15^N values as a result of ethanol preservation (Table 1, Fig. 2).

**Table 1:** Comparison of isotopic values (means ± SD) of 24 loggerhead egg yolk samples that underwent different treatments

Main effect	Isotope	Treatment		Difference	*P* Value
		Frozen	Ethanol		
Preservative	δ^13^C (‰)	−19.59 ± 1.20	−19.33 ± 1.22	−0.26 ± 0.02	N/A
	δ^15^N (‰)	12.86 ± 1.44	12.75 ± 1.39	−0.13 ± 0.05	0.053
		Whole	Lipid extracted		
Lipid content	δ^13^C (‰)	−20.21 ± 1.22	−18.70 ± 0.97	1.51 ± 0.25	N/A
	δ^15^N (‰)	12.74 ± 1.39	12.87 ± 1.44	0.13 ± 0.05	**0.036**

The effects of preservative (frozen and ethanol preserved) and lipid extraction (whole and lipid extracted) were tested using a mixed-effects model. As there was a significant interaction effect of preservative and lipid extraction on δ^13^C values, the main effects could not be interpreted independently (N/A). Further analysis using Tukey's *post hoc* HSD test was completed for δ^13^C values as pair-wise group comparisons (see Table 2). Significant *P* values are bold.

**Table 2:** Pair-wise differences in δ^13^C values (expressed as parts per thousand) among loggerhead yolks in four different treatment combinations

	Frozen, whole	Frozen, lipid extracted	Ethanol, whole	Ethanol, lipid extracted
Frozen, whole				
Frozen, lipid extracted	1.02 (** < 0.001**)			
Ethanol, whole	−0.24 (0.12)	−1.25 (** < 0.001**)		
Ethanol, lipid extracted	1.77 (** < 0.001**)	0.75 (** < 0.001**)	2.00 (** < 0.001**)	

Values in parentheses are *P*?values, and bold values are significant. All comparisons were significant except between frozen, whole yolks and ethanol-preserved, whole yolks.

**Table 3:** Linear relationships between δ^13^C values in 24 loggerhead egg yolks that underwent different treatments as well as the change in δ^13^C values (Δδ^13^C) after lipid extraction using the whole yolk C:N ratios

	δ^13^C	C:N vs. Δδ^13^C
Effect of preservative		
Whole yolk	No correction necessary	N/A
Lipid-extracted yolk	*δ*^13^C_F_ = 1.11 × *δ*^13^C_E_ + 1.18 (*r*^2^ = 0.91)	N/A
Effect of lipid extraction		
Frozen yolk	δ^13^C_LE_ = 0.76 × δ^13^C_W_ − 3.83 (*r*^2^ = 0.91)	Δδ^13^C = 0.49 × C:N_W_ −1.65 (*r*^2^ = 0.46)
Ethanol-preserved yolk	δ^13^C_LE_ = 0.62 × δ^13^C_W_ − 5.83 (*r*^2^ = 0.82)	Δδ^13^C = 0.54 × C:N_W_ − 1.14 (*r*^2^ = 0.50)

Abbreviations: E, ethanol preserved; F, frozen; LE, lipid extracted; and W, whole (not lipid extracted). All reported regressions were significant with *P* < 0.001.

Lipid extraction significantly increased δ^13^C values of loggerhead egg yolks, regardless of preservation method, as indicated by the Tukey's *post hoc* comparisons. Frozen yolk δ^13^C values increased by a mean of 1.02‰ (Table 2), while ethanol-preserved yolk δ^13^C values increased by a mean of 2.00‰ (Table 2). The effect of lipid extraction on the δ^13^C values of frozen and ethanol-preserved samples can be calculated using the normalization equations in Table 3. The main effect of lipid extraction on the δ^15^N values of yolk was significant (Table 1), but the mean change of 0.14‰ in δ^15^N values was less than the standard deviation for the laboratory reference material, loggerhead turtle scute (0.28‰), and is therefore not likely to be significant biologically. Therefore, we do not provide normalization equations for the effect of lipid extraction on δ^15^N values.

**Figure 3:**
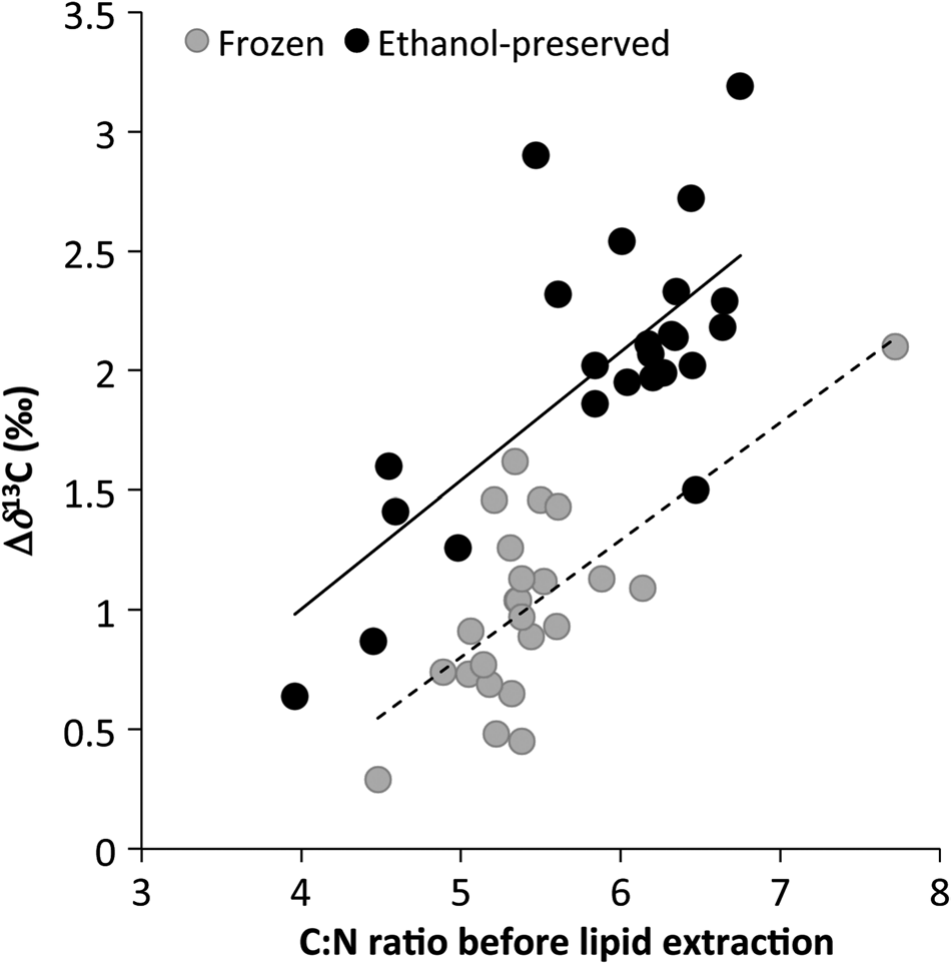
The change in δ^13^C values following lipid extraction (Δδ^13^C) is positively related to the C:N ratio prior to lipid extraction in both frozen and ethanol-preserved yolks. Both relationships are significant (*P* < 0.001), and equations are provided in Table 3.

Mean C:N ratios decreased significantly after lipid extraction with both preservation methods (Table 4). The linear regressions between whole yolk C:N and Δδ^13^C values were significant (Table 3, Fig. 3). However, given the lower *r*^2^ values (0.46 for frozen yolks and 0.50 for ethanol-preserved yolks) of using C:N ratios compared with the higher *r*^*2*^ values (0.91 for frozen yolks and 0.82 for ethanol-preserved yolks) of using the δ^13^C values for correcting for the effects of lipid extraction, we do not recommend using the C:N ratios for prediction.

**Table 4:** Comparison of C:N ratios of whole and lipid-extracted (LE) yolks with different methods of preservation (means ± SD)

	Whole yolk C:N	LE yolk C:N	Difference	*P* Value
Frozen yolk	5.44 ± 0.58	4.02 ± 0.06	−1.42 ± 0.58	** < 0.001**
Ethanol-preserved yolk	5.86 ± 0.79	3.48 ± 0.14	−2.38 ± 0.67	** < 0.001**

### Mother–egg relationship

**Figure 4:**
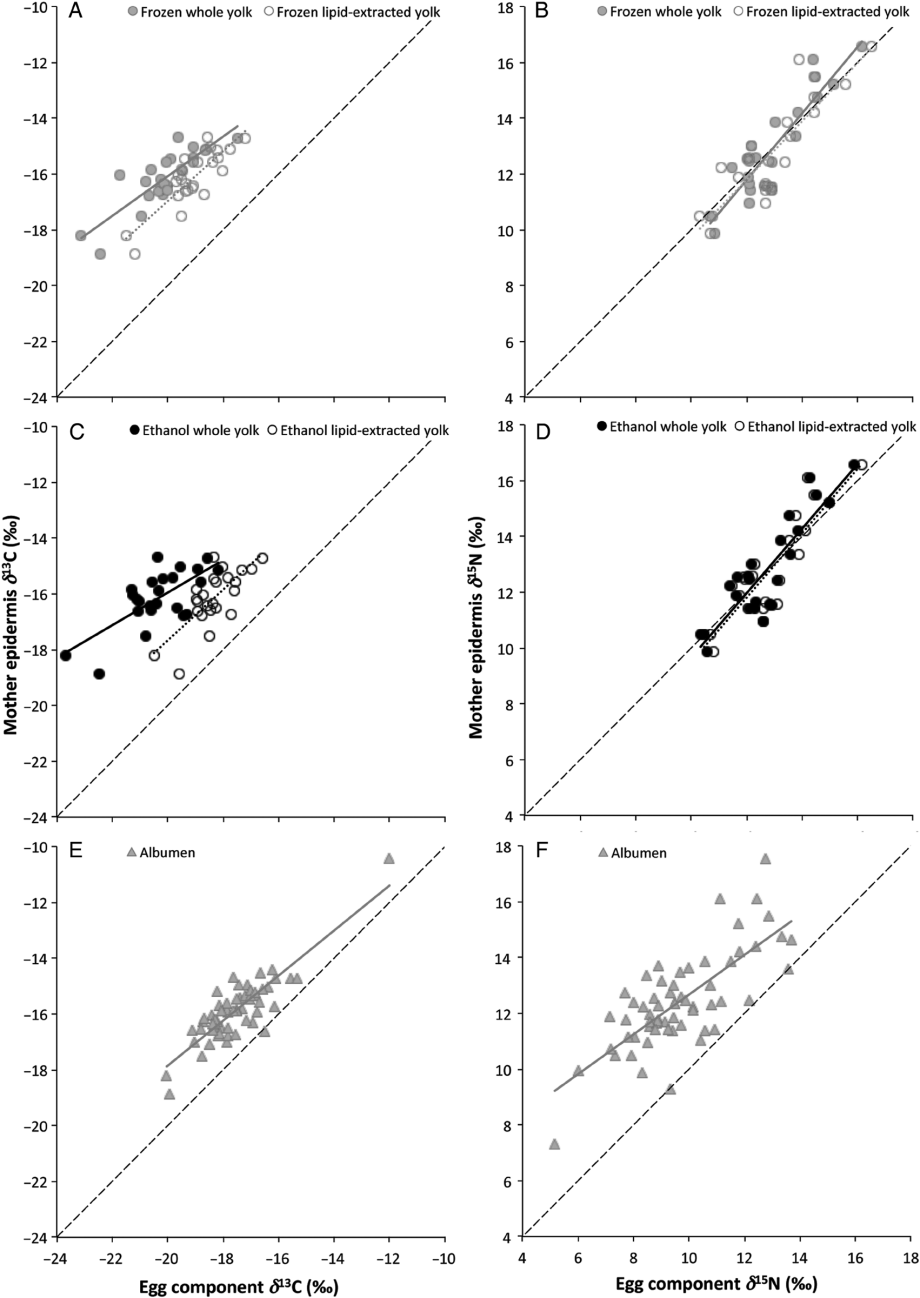
Linear relationships between maternal epidermis and egg component δ^13^C (**A**, **C** and **E**) and δ^15^N values (**B**, **D** and **F**). Four different yolk treatments (A–D; *n* = 24) as well as albumen (E and F; *n* = 61) were examined. All relationships shown are significant (*P* < 0.001), and equations are provided in Table 5. The length of the regression line corresponds to the range of stable isotope values measured in the present study. A one-to-one line is provided for reference in each panel.

The linear relationships between mother and offspring were determined by using stable isotope values from maternal epidermis samples and corresponding egg components, i.e. yolk (*n* = 24 for each of four treatments) as well as albumen (*n* = 61). For all four of the yolk treatment combinations, δ^13^C values were lower than those of materanl epidermis, while for all yolk treatments (except for frozen, whole yolks) δ^15^N values were more similar to maternal epidermis (Fig. 4). Albumen samples had lower δ^13^C and δ^15^N values than maternal epidermis samples (Fig. 4). Significant linear regression equations for both δ^13^C and δ^15^N values may be used to approximate maternal isotopic values from egg yolk and albumen (Table 5).

**Table 5:** Linear relationships to predict loggerhead turtle maternal epidermis (epi) δ^13^C and δ^15^N values using corresponding egg components

Egg component	*n*	Maternal predicted δ^13^C (‰)	Maternal predicted δ^15^N (‰)
F, W yolk	24	δ^13^C_epi_ = 0.71 × *δ*^13^C_yolk_ − 1.86	δ^15^N_epi_ = 1.19 × δ^15^N_yolk_ − 2.50
		(*r*^2^ = 0.70)	(*r*^2^ = 0.82)
F, LE yolk	24	δ^13^C_epi_ = 0.90 × *δ*^13^C_yolk_ − 0.95	δ^15^N_epi_ = 1.05 × δ^15^N_yolk_ − 0.75
		(*r*^2^ = 0.70)	(*r*^2^ = 0.72)
E, W yolk	24	δ^13^C_epi_ = 0.58 × δ^13^C_yolk_ − 4.27	δ^15^N_epi_ = 1.16 × δ^15^N_yolk_ − 1.90
		(*r*^2^ = 0.47)	(*r*^2^ = 0.77)
E, LE yolk	24	δ^13^C_epi_ = 0.91 × δ^13^C_yolk_ + 0.53	δ^15^N_epi_ = 1.15 × δ^15^N_yolk_ − 2.04
		(*r*^2^ = 0.53)	(*r*^2^ = 0.76)
Albumen	61	δ^13^C_epi_ = 0.81 × δ^13^C_alb_ − 1.73	δ^15^N_epi_ = 0.72 × δ^15^N_alb_ + 5.55
		(*r*^2^ = 0.74)	(*r*^2^ = 0.56)

Abbreviations: E, ethanol preserved; F, frozen; LE, lipid extracted; and W, whole (not lipid extracted). All regressions were significant with *P* < 0.001 and are plotted in Fig. 4.

### Foraging area assignment accuracy

The equations from Table 5 were used to convert loggerhead egg yolk and albumen samples to maternal epidermis stable isotope values to assess the reliability of determining the foraging area origin of the mother by using converted egg components as a proxy. The accuracy of assignment using egg component isotopic values ranged from 77 to 84% (Table 6). The lowest accuracy of assignment (77%) was found using ethanol-preserved, whole yolk samples, whereas the highest (84%) was obtained using albumen samples. Not all yolks could be assigned with a probability ≥80%. Yolks that were preserved in ethanol and lipid extracted resulted in the lowest proportion of assignments (14 of 20), while yolks that were frozen and whole resulted in the highest proportion of assignments (20 of 20). Therefore, it is important to consider both the proportion of assignments as well as their accuracy.

**Table 6:** Comparison of foraging area assignment accuracy using the original maternal epidermis stable isotope values and converted egg components

Sample type	Mothers assigned	Number of egg samples assigned	Number of egg samples unassigned	Foraging area			Assignment accuracy (%)
				MAB	SAB	SNWA	
F, W yolk	20	20	0	14 (3)	2 (1)	0	80.0
F, LE yolk	20	18	2	14 (2)	1 (1)	0	83.3
E, W yolk	20	17	3	13 (3)	0 (1)	0	76.5
E, LE yolk	20	14	6	11 (2)	0 (1)	0	78.6
Albumen	49	45	4	32 (7)	5 (0)	1 (0)	84.4

Assignments were first performed using maternal epidermis values. The ‘mothers assigned’ column represents the number of maternal epidermis samples that could be assigned to one of three foraging areas with probability of group membership ≥80%. Then stable isotope values of loggerhead egg components (yolk with four treatments and albumen), for which the maternal epidermis met the assignment threshold, were converted to maternal epidermis values using the equations in Table 4 and assigned to a foraging area. The number of egg samples assigned represents the number of converted sample assignments that resulted in a probability of group membership ≥80%, while those falling below the probability threshold were classified as unassigned egg samples. The ‘foraging area’ columns show the distribution of assignments, and values in parentheses are the number of converted egg samples assigned to that foraging area that did not match the assignment using the maternal epidermis. Egg yolk treatments were as follows: E, LE = ethanol preserved and lipid extracted; E, W = ethanol preserved, whole; F, LE = frozen and lipid extracted; and F, W = frozen whole. The foraging areas are as follows: MAB, Mid-Atlantic Bight; SAB, South Atlantic Bight; and SNWA, Subtropical Northwest Atlantic.

## Discussion

### Effect of ethanol preservation

Differential effects of ethanol were observed for the δ^13^C values of egg yolks (that were whole and lipid extracted). Investigating the mechanistic cause for the differential effects was beyond the scope of this study, but the C:N ratios may provide a potential explanation. Although C:N ratios are typically representative of lipid content, the C:N ratio may not be reflecting a higher lipid content in this case, but rather an effect of the preservative. The polar, ethanol preservative may be contributing to the extraction of additional lipids and other carbon-containing compounds in the lipid extraction process. Before lipid extraction, yolks preserved in ethanol had a higher average C:N ratio than frozen yolks (Table 4). After lipid extraction, yolks preserved in ethanol had a lower average C:N ratio than frozen yolks (Table 4). In these conditions, ethanol-preserved yolks exhibit higher δ^13^C values than frozen yolks (Fig. 2A). The variations in C:N ratios suggest that lipid extraction causes a larger change in lipid content of ethanol-preserved yolks than frozen yolks. In addition to lipid extraction, the polar preservative (ethanol) may be depleting the sample of carbon-containing compounds, as seen in lipid extraction using polar solvents (Dobush *et al.*, 1985; Doucette *et al.*, 2010). This could consequently be contributing to the lower δ^13^C values that were observed in the ethanol-preserved whole yolks. Therefore, the opposite changes observed in δ^13^C values in the two different ethanol treatments were likely to result from the differential effects of this preservative prior to and during lipid extraction.

Although we expected that preservation in 95% ethanol would have no effect on stable isotope values of egg components, two previous publications indicated that preservation in 95% ethanol decreased δ^13^C values of samples that were not lipid extracted (Ponsard and Amlou, 1999; Kelly *et al.*, 2006). These same studies did not find a significant effect of ethanol preservation on δ^15^N values (Ponsard and Amlou, 1999; Kelly *et al.*, 2006), thus resembling the results of the present study.

Given that ethanol preservation significantly affected δ^13^C values of egg yolk when lipid extracted, freezing samples may be a preferable method of preservation. Under circumstances in which freezing is not a feasible method of preservation to use in the field, the isotopic effects of ethanol can be reliably corrected for in loggerhead yolk samples that are preserved this way. The linear regressions between the δ^13^C values of frozen and ethanol-preserved yolks that are lipid extracted explain a high amount of variation. We expect that ethanol preservation would result in similar effects for eggs of other marine sea turtle species. However, because the studies previously discussed show contradictory effects of ethanol preservation on samples, we would caution against using these equations for other tissues or taxa.

### Effect of lipid extraction

Lipid extraction was found to increase yolk δ^13^C and δ^15^N values significantly for both frozen and ethanol-preserved yolks. Values of δ^13^C were affected more substantially in ethanol-preserved samples than frozen samples (Table 2). Yolk δ^13^C values were expected to increase significantly after lipid extraction, because lipids have lower δ^13^C values than other cell components (DeNiro and Epstein, 1977). For both ethanol-preserved and frozen yolks, the change in δ^13^C values after lipid extraction corresponded to expected decreases in C:N ratios (Table 4).

The significant change in δ^15^N values following lipid extraction with a non-polar solvent was not expected. While lipid extraction with polar solvents commonly causes increases in δ^15^N values (Sweeting *et al.*, 2005; Doucette *et al.*, 2010; Oppel *et al.*, 2010a), non-polar solvents do not tend to affect δ^15^N values (Doucette *et al.*, 2010). The cited studies used various tissues (muscle, liver and yolk) from multiple species. The effect of non-polar solvents may be dependent on tissue type and species because an increase in mean δ^15^N values after lipid extraction using non-polar petroleum ether was observed in homogenized eggs from seven species of aquatic birds (Elliott *et al.*, 2014). Although the change in δ^15^N values after lipid extraction in this study was in the same direction as that of Elliott *et al.* (2014), the mean change in loggerhead eggs was much lower (0.1 vs. 1.0‰), and this change was less than the standard deviation for the laboratory reference material, loggerhead turtle scute (0.28‰). Therefore, this observed change in nitrogen isotopic values is not likely to be significant biologically, and we do not provide linear regressions to correct for the effects of lipid extraction on δ^15^N values.

It has been suggested that samples from aquatic animals with a C:N ratio >3.5 have a lipid content that is too high to obtain reliable δ^13^C values, and thus, lipid extraction or normalization is recommended (Post *et al.*, 2007). Although the C:N of all of the yolks decreased after lipid extraction regardless of preservation method, the resulting C:N ratios of frozen yolk were still higher than what is thought to be acceptable for reliable δ^13^C values (Table 4). The average C:N after lipid extraction was >4.0 for frozen yolks. Pure protein may not always have a C:N ratio close to 3.0 due to variations in amino acid composition (Elliott *et al.*, 2014); therefore, the C:N cut-off recommended by Post *et al.* (2007) may not apply to all situations. Different solvents may remove different lipid components (Elliott *et al.*, 2014), and it is also possible that the high C:N ratios observed in the present study were a result of incomplete lipid removal. A negative correlation between C:N ratios and δ^13^C values would suggest a bias due to incomplete lipid removal, but the lack of a relationship between these factors in our data suggests that lipid removal was complete in both frozen and ethanol-preserved yolks.

Lipid normalization using C:N ratios has been used reliably to predict the δ^13^C values of lipid-extracted muscle tissue in a wide variety of organisms (Post *et al.*, 2007; Ehrich *et al.*, 2011) but has been more problematic in avian eggs (Ricca *et al.*, 2007; Oppel *et al.*, 2010a; Ehrich *et al.*, 2011; Elliott *et al.*, 2014), possibly due to the high lipid content of avian yolks, which can contain >50% lipids and have C:N ratios as high as 20.2 (Oppel *et al.*, 2010a; Ehrich *et al.*, 2011). Likewise, our attempts to relate the C:N ratio of loggerhead egg yolks to Δδ^13^C values (the change in δ^13^C values as a result of lipid extraction) resulted in linear regressions with low *r*^2^ values (0.46 for frozen yolks and 0.50 for ethanol-preserved yolks); therefore, we do not recommend using C:N ratios as a correction method. The weak correlation observed in the present study may also be due to the high lipid content of loggerhead turtle egg yolks prior to lipid extraction, which have been measured to contain up to 25% lipids (Alava *et al.*, 2006) and a mean C:N ratio of 5.4 (present study).

Instead, the stable isotope values of lipid-free yolks can be predicted reliably by measuring stable isotope values in whole yolks and then applying the linear regressions from the present study. These equations account for much of the variation observed between the δ^13^C values of whole and lipid-extracted yolks (*r*^2^ values >0.82; Table 3). Lipid normalization using these equations in future studies can avoid the process of lipid extraction, which can be time consuming and can potentially involve hazardous chemicals. The lipid content of other marine turtle eggs is likely to be similar to that of loggerhead eggs, and therefore, we expect that these equations could be used reliably for lipid normalization in other marine turtle species.

### Mother–egg relationship

The linear regressions provided in the present study permit the approximation of maternal epidermis δ^13^C and δ^15^N values from both yolk and albumen, as they described statistically significant correlations. Yolks (in all treatment combinations) and albumen had consistently lower δ^13^C values in comparison to maternal epidermis values. This effect is likely to be due to the high lipid content of energy-rich eggs (Bouchard and Bjorndal, 2000; Alava *et al.*, 2006), although δ^13^C values in yolk, even after lipid extraction, were lower than those of maternal epidermis. We expected an increase in egg δ^15^N values relative to maternal epidermis because eggs are derived from maternal nutrients. Mother–offspring differences in tissue δ^15^N are thought to be similar to the difference between diet and tissue δ^15^N values (Jenkins *et al.*, 2001; Sare *et al.*, 2005; McMeans *et al.*, 2009), which is typically of the order of 3–4‰ (Post, 2002). However, this pattern was not observed between maternal epidermis and egg components. Albumen had lower δ^15^N values (differences as much as −5‰), whereas yolk had slightly higher δ^15^N values (differences less than +1‰) when compared with maternal epidermis. Examinations of avian yolk and albumen in comparison to δ^15^N values in the maternal diet have found that egg component δ^15^N values are typically higher than those of the mother's diet (Hobson, 1995; Polito *et al.*, 2009; Federer *et al.*, 2012). There are no isotopic comparisons of egg albumen with maternal tissue in the literature, and we are uncertain why albumen δ^15^N values were lower than those of maternal epidermis in the present study.

Previous studies have demonstrated that carbon and nitrogen stable isotope values of loggerhead female epidermis are correlated with those of hatchling epidermis and unhatched eggs (Frankel *et al.*, 2012; Ceriani *et al.*, 2014). The mother–hatchling epidermis relationships for both δ^13^C and δ^15^N values were significant (Frankel *et al.*, 2012), but the mother–hatchling δ^13^C correlation (*r*^2^ = 0.24) was much lower than the mother–egg relationships observed in the present study (Table 5). Therefore, yolk or albumen isotopic values may be a more reliable proxy than hatchling epidermis for maternal epidermis. Isotopic conversions between tissue types are consistent among many bird species (Cherel *et al.*, 2014), and therefore, the relationship between mother and offspring would be expected to be conserved among different loggerhead populations as well as different sea turtle species. However, comparing the mother–unhatched egg conversion equations from Ceriani *et al.* (2014) with the frozen, lipid-extracted yolk conversions in the present study reveals large differences between the equations (present study, δ^13^C_epi_ = 0.90δ^13^C_yolk_ – 0.95, δ^15^N_epi_ = 1.05δ^15^N_yolk_ − 0.75; and Ceriani *et al.*, 2014, δ^13^C_epi_ = 1.07δ^13^C_egg_ + 3.64, δ^15^N_epi_ = 1.14δ^15^N_egg_ − 2.47). These differences may be a result of the different tissue types (fresh egg yolks vs. entire unhatched eggs), and therefore, caution may be needed in applying conversion equations to situations without equivalent sample types.

There are trade-offs between collecting different sample types, as fresh eggs must be sacrificed, while skin can be collected from live hatchlings without significantly affecting their health or physiological status (Bjorndal *et al.*, 2010). The stable isotopic composition of unhatched eggs is reliably correlated with the isotopic composition of fresh egg yolks, and thus, it may be possible to avoid sacrificing an egg (Ceriani *et al.*, 2014). Nevertheless, clutches may be lost during incubation due to stochastic events, such as storms and predation, and thus, collecting a fresh egg guarantees that a sample can be obtained from a particular clutch.

### Foraging area assignment accuracy

The goal of providing the mother–egg conversion equations is to predict the female isotope values from egg components when a female is not encountered. Regular sampling of a nesting population for stable isotope values can be valuable to monitor trends in the abundance of females originating from distinct foraging areas (Vander Zanden *et al.*, 2014a). We applied the conversions that were determined in the present study to calculate maternal epidermis values from yolk and albumen samples in order to determine the mother's foraging area. We found that these egg components can be used to predict the foraging location of the mothers reliably, although accuracy and the percentage of assignments that met our probability threshold (≥80%) varied among treatments. Nevertheless, the high level of accuracy (>76%) in predicting the mother's foraging area from all egg components supports the use of these equations when using loggerhead egg tissue stable isotope values as a proxy for those of the mother. The highest level of accuracy was achieved by converting albumen isotopic values, followed closely by frozen, lipid-extracted yolk and frozen, whole yolk. Therefore, we recommend using frozen egg yolk (whole or lipid extracted) or albumen samples to assign a large proportion of individuals correctly, when possible. If freezing tissues is not an option, ethanol-preserved egg yolks are also a reliable proxy for maternal isotopic values.

### Conclusions

Preservation in 95% ethanol and lipid extraction significantly altered loggerhead egg yolk δ^13^C composition. Despite the interactive effect of these two treatments, by using the treatment-specific mathematical corrections provided here, future studies can account for effects of ethanol preservation and lipid extraction on sea turtle yolk stable isotope values. No correction is necessary for δ^15^N values, because they were minimally affected by each treatment. With the δ^13^C normalization equations, researchers can preserve egg yolk samples in ethanol when freezers are not accessible and bypass the time-consuming process of lipid extraction. In addition, maternal isotopic values can be approximated from egg components as a result of the strong correlation between maternal epidermis and either yolk or albumen. We verified that these converted values can be used to predict female foraging area in the NWA with high accuracy. Future studies will be able to investigate loggerhead foraging locations and trophic relationships without having to encounter and sample nesting females. This ecological knowledge is vital to the protection of endangered loggerhead turtles, because successful conservation strategies must account for the global distribution of sea turtles.

## Funding

This work was supported by the National Fish and Wildlife Foundation; National Marine Fisheries Service; and United States Fish and Wildlife Service.
